# The Role of Molecular Imaging in a Muscle-Invasive Bladder Cancer Patient: A Narrative Review in the Era of Multimodality Treatment

**DOI:** 10.3390/diagnostics11050863

**Published:** 2021-05-11

**Authors:** Vincenzo Cuccurullo, Giuseppe Danilo Di Stasio, Francesco Manti, Pierpaolo Arcuri, Rocco Damiano, Giuseppe Lucio Cascini

**Affiliations:** 1 Nuclear Medicine Unit, Department of Precision Medicine, Università della Campania “Luigi Vanvitelli”, 81100 Napoli, Italy; 2 Nuclear Medicine Division, European Institute of Oncology, 20144 Milan, Italy; d.distasio91@gmail.com; 3 Nuclear Medicine Unit, Department of Diagnostic Imaging, Magna Graecia University of Catanzaro, 88100 Catanzaro, Italy; manti@unicz.it (F.M.); arcuri@unicz.it (P.A.); cascini@unicz.it (G.L.C.); 4 Unit of Urology, Magna Graecia University, 88100 Catanzaro, Italy; damiano@unicz.it

**Keywords:** bladder cancer, nuclear medicine, diagnostic imaging, restaging, response evaluation, PET/CT, hybrid imaging

## Abstract

Diagnostic imaging in bladder cancer plays an important role since it is needed from pretreatment staging to follow-up, but a morphological evaluation performed with both CT and MRI showed low sensitivities and specificities in detecting pathologic lymph nodes, due to the occurrence of false positive results. Implementation of functional information provided by PET/CT could be a determinant in the management of patients with muscle-invasive bladder cancer. A focus on the role of ^18^F-FDG PET/CT and alternative tracers in patients with muscle-invasive bladder cancer is provided in this analysis in order to outline its potential applications in staging settings and response evaluation after neoadjuvant chemotherapy.

## 1. Introduction

Bladder cancer (BC) represents the sixth most common malignancy in the United States [1] and accounts for approximately 430,000 new cases and over 165,000 deaths/year, with a three to four times higher incidence in men than in women [2]. Clinical management of BC is challenging due to the heterogeneity of tumors [3], in terms of loco-regional invasion and metastasis, and due to the presence of environmental factors such as polycyclic aromatic hydrocarbons (PAH), aromatic amines (AA), nitrosamines, cigarette smoking, occupational exposure, and hair dyes, rather than infections, like schistosomiasis, which results in squamous cell bladder carcinoma [4]. The most common form of BC (more than 90% of the cases) in developed countries is urothelial carcinoma, derived from the uroepithelium that covers the bladder, whereas the remaining are less common types, such as squamous cell carcinomas, adenocarcinomas, small cell carcinomas, and sarcomas.

The clinical management of patients with BC is a complex and not well standardized process, in which multiple options may be selected from the diagnosis to final treatment [5]. Therefore, significant shifts are required to define the best options in diagnosis and treatment for BC [6] and also to incorporate molecular medicine with the final aim of decreasing ineffective or inappropriate treatments in any single patient [7].

Molecular nuclear medicine of the urinary tract focuses on conventional imaging techniques of the kidney [8]. In past years, bladder imaging has mainly been limited to the identification of vesicoureteral reflux as a cause of recurrent urinary tract infections [9,10].

The introduction of positron emission tomography combined with computed tomography (PET/CT) in the scenario of molecular medicine has represented a new opportunity in the setting of patients with bladder cancer, although many technical drawbacks negatively affect diagnostic performances [11]. The intense urinary excretion of ^18^F-Fluorodeoxyglucose (^18^F-FDG) [12], for example, highly reduces the sensitivity of the technique for diagnostic purposes; nonetheless, PET/CT is still superior to conventional morphological imaging for detecting nodal involvement and recurrence, especially if some precautions are used, such as delayed imaging, fluid loading, diuresis or bladder catheterization [13]. A focus on the role of ^18^F-FDG PET/CT in patients with muscle-invasive bladder cancer is provided in this study, trying to outline its potential applications in both staging and restaging settings and also for response evaluation following neoadjuvant chemotherapy (NAC).

## 2. Physiopathological Premises and Current Decisional Algorithm

Urothelial BC is classified as a non-muscle invasive bladder cancer (NMIBC)—cancer which has not invaded through the smooth muscle layer surrounding the bladder, representing the majority of BC diagnoses (roughly 75%) [4]. Muscle-invasive bladder cancer (MIBC) tends to be already metastatic at initial presentation and is associated with a predictable pattern of pelvic and iliac lymph node metastases in visceral sites, most commonly lung, liver, and bone [5]. Accurate staging of bladder cancer is mandatory to select the appropriate treatment strategy, as invasive bladder cancer when metastatic to other sites is rarely curable [14]. From a physiopathological point of view, MIBC is linked to several risk factors and different molecular pathways that need to be better understood in order to improve the diagnosis and treatment of such patients [15]

In addition, invasive tumors are characterized by a high overall mutation rate and chromosomal aberrations, with mutations that are mainly mediated by APOBEC mutagenesis, drawing in the most important cellular pathways, including p53, Rb, PI3K-mTOR, and RAS [16]. Alterations in cadherins, vascular endothelial growth factors (VEGFs), matrix metalloproteinases (MMPs), and thrombospondin-1(TSP-1), i.e., factors involved in remodeling the extracellular matrix and that promote tumor angiogenesis, are more common in MIBC (T2–T4) and also contribute to nodal metastasis, with post-cystectomy recurrences that are higher in patients with MIBC and with a poorer prognosis [17]. The involvement of one pathway more than the others determines the identification of the so-called expression profile, which led to the division in two main subtypes of BCs, namely basal and luminal [18]. The former are less differentiated tumors, highly enriched with p63 activation, which tend to be more aggressive and lethal with a squamous differentiation; whereas the latter are more differentiated and are characterized by activating FGFR3 mutations, since mutations in HRAS and FGFR3 decrease with invasion, while the opposite happens for p53, p21, Rb, and p16 alterations [19]. These findings demonstrate the high tumor heterogeneity in patients affected by MIBC, with significant differences in terms of clinical expression of disease as well as treatment response and final outcome.

All these biological aspects assume relevance in terms of prognosis, as demonstrated by Robertson et al., who observed four signature clusters with different 5-year survival probabilities, ranging between 75% in high APOBEC mutational load and 22% of MIBC with lower mutations [20].

BC is usually detected after the clinical observation of hematuria, which could be either microscopic or gross [21]. The gold standard in diagnosis and follow-up of BC is cystoscopy, which has a high sensitivity and specificity, up to 95% and 100%, respectively, along with high positive predictive value (PPV) and negative predictive value (NPV) depending on the prevalence of the disease in the population [22]. However, limitations involve flat lesions and papillary lesions that could be missed and there is no assessment of nodal and extravesical involvement [21,22]. Therefore, imaging of the upper tract and pelvis, usually with conventional imaging techniques, i.e., CT and/or MRI, is performed in parallel to appropriately stage all patients and address them to direct surgery rather than neoadjuvant cisplatin-based chemotherapy (NAC) [23]. In fact, for patients with MIBC, the standard of care treatment consists of platinum-based NAC, followed by radical cystectomy (RC) with bilateral pelvic lymphadenectomy, since RC alone is associated with unacceptably high recurrence rates, mostly due to distant disease and poor prognosis (5-year OS up to 50%) [24]. Thus, the use of NAC represents an attempt to eliminate the presence of micro-metastatic disease before surgery, and in this sense patients with MIBC who underwent NAC showed a downgrade of disease and an overall significantly improved prognosis [25]; in particular, as a recent metanalysis demonstrated, NAC provided a 5% absolute OS improvement and a disease-free survival improvement of 9% at 5 years [26].

However, high grade toxicities have been reported in more than one patient out of three in SWOG and EORTC studies [27] and these data are not always balanced by significant improvement in OS in MIBC patients; thus, the selection of patients with clinical T3 and T4 that may benefit from NAC without toxicities is the challenge for modern oncology and diagnostic imaging in the future. According to this scope, MIBC could be further divided into low and high risk, which includes ≥cT3b disease; presence of hydroureteronephrosis; lymphovascular invasion; or more aggressive variant histology, such as squamous, sarcomatoid, etc. [28].

All these data support the need for a holistic vision of patients with MIBC, taking into account mutational status, histological assessment, and TNM staging. The latter still persists as independent prognostic factor since 5-year relative survival rate for carcinoma in situ is 96%, for localized disease is 70%, regional disease is 36%, and distant disease is 5% [29]. For these reasons, diagnostic imaging is pivotal for an appropriate TNM staging in clinical practice.

## 3. Role of Diagnostic Imaging

In this context, diagnostic imaging plays an important role, since it is needed from pretreatment staging to follow-up. In particular, as stated in the latest American College of Radiology Appropriateness Criteria report [30], pretreatment staging of MIBC should include imaging of the urothelial upper tract for synchronous lesions with MRI of the pelvis for local staging and/or CT of the abdomen and pelvis without and with contrast (CT urogram) to assess the urothelium and abdominopelvic organs, plus imaging of the chest, abdomen, and pelvis for metastases. Unfortunately, the morphological evaluation performed with both CT and MRI showed low sensitivities and specificities in detecting pathologic lymph nodes, due to the occurrence of false positives results [31]. They are possible because the main criteria used to deem a lymph node as suspicious for metastatic disease is dimensional, with current recommendations that suggest a cut-off of ≥8 mm for pelvic lymph nodes and ≥1 cm for retroperitoneal ones [32]. This could lead to an understaging of metastatic disease in up to 30% of patients. In fact, despite new MRI sequences, such as diffusion-weighted and dynamic contrast-enhanced imaging, that may improve our ability of nodal staging, [33,34,35] MRI and CT have been shown to perform similarly because both techniques rely on morphological information while lacking metabolic data [36].

In this sense, the implementation of functional information provided by nuclear medicine thanks to PET/CT could be a determinant in the management of patients with MIBC [37].

## 4. ^18^F-FDG PET/CT for Preoperative Lymph Node Staging of MIBC

**Figure 1:**
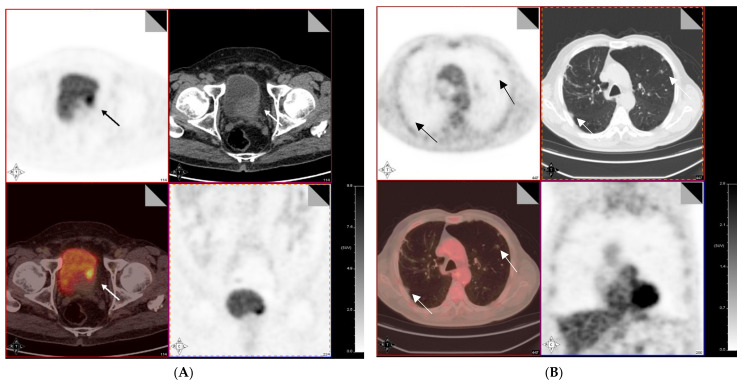
Transaxial FDG-PET with hydration (upper left), CT (upper right), fused (lower left), and MIP (lower right) of a patient with MIBC during staging before treatment. Focal intense FDG uptake on the left postero-lateral wall (solid arrow), without any other pathological uptake out of the bladder (**A**). In particular, small bilateral lung nodules did not show significant metabolism (**B**). The patient was submitted to cystectomy with wide lymphectomy, demonstrating pT4N0 MIBC. Lung nodules still persist as stable in the following TC.

**Figure 2:**
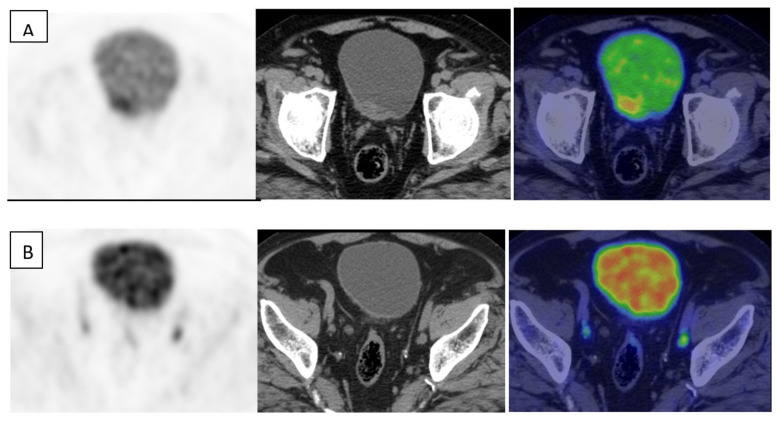
Transaxial FDG-PET with hydration (left), CT (center), and fused (right) images of MIBC in the posterior wall. Intense glucose metabolism in the primary tumor higher than FDG urinary excretion is present (**A**). CT images show bilateral iliac lymph nodes enlargement corresponding to focal areas of glucose uptake (**B**). Histology confirmed iliac lymph node involvement.

In this section, we analyze the role of PET with different technical approaches in MIBC, excluding primary tumor detection, where cystoscopy, CT, and MRI have demonstrated a prominent role, although MIBC may be FDG avid (Figure 1 and Figure 2) with intense uptake. However, the main incremental value of PET during staging MIBC is the assessment of lymph node status and detection of distant metastases; PET is widely used to address this issue. Although, only one not updated meta-analysis showed a pooled sensitivity of 0.82 and specificity of 0.89 for metastatic lesion detection [38].

^18^F-FDG PET/CT is the most common molecular imaging technique for preoperative staging of various malignancies, due to the higher metabolic rate of cancerous cells compared to normal ones [39,40,41,42], however, as a glucose analog, ^18^F-FDG is excreted in urine and therefore it physiologically accumulates in the bladder, thus eventually masking the uptake of any superficial wall lesions and/or of the adjacent lymph nodes [43].

In a 2019 prospective study of 35 patients with MIBC [44], the authors focused on the possible use of delayed post-diuretic ^18^F-FDG PET/CT in both staging (18 patients) and restaging (17 patients) scenarios; in the former group, thanks to delayed post-diuretic image acquisition, seven patients were upstaged with consequent changing in therapeutic management, whereas in the latter, responses were documented in eleven patients where they proceeded to radical surgery. The imaging protocol involved an intravenous injection of 20 mg of furosemide immediately after the acquisition of whole-body PET/CT data, followed by 0.8–1 L oral water hydration; patients were also informed to void frequently and then to hold urine to allow maximum bladder distension. In fact, thanks to the diuretic, the excretion rate increased with a rapid washout and clearance of urine activity, which occurred after 30–60 min. Pelvic PET/CT images were then acquired using the same parameters about 1 h after diuretic injection. Therefore, they concluded that delayed post-diuretic ^18^F-FDG PET/CT helped in the determination of the best treatment decision in 68.6% of patients among both groups, thus representing an important diagnostic tool in the evaluation of MIBC patients. In this sense, the latest American College of Radiology Appropriateness Criteria lists ^18^F-FDG PET/CT in the category of “may be appropriate” for BC staging [30], since it improves sensitivity, especially if combined with CT, in the case of muscle-invasive forms in which nodal and extravesical involvement is highly suspected and for the assessment of subcentimetric nodes where the traditional dimensional criterion cannot be of help [30]. In fact, using some precautions before image acquisition could significantly improve the sensitivity of the technique [45,46]. For instance, Uttam et al. [47] studied fifteen patients with MIBC undergoing RC with ^18^F-FDG PET/CT to assess the role of the technique for the preoperative lymph node staging; to improve the diagnostic performance they administered 500 mL normal saline and 20 mg of furosemide to all patients 10–15 min after FDG injection and patients were also asked to void frequently in order to avoid accumulation of FDG metabolite in the urinary bladder, which causes difficulty in the analysis of lymph nodes. They reported 100% sensitivity, but with a low specificity of 58.3%, thus concluding that it inevitably represents a major concern for its use in clinical practice, since physicians have to decide whether to go ahead with RC or not in patients with positive nodes. In another study of 233 patients with either MIBC or high-risk non-MIBC in which researchers considered potential candidates for radical cystectomy, Goodfellow et al. [48] found that when using ^18^F-FDG PET/CT, the sensitivity for pelvic lymph node involvement, compared to CT alone, increased from 45% to 69% with a slight decrease in specificity from 98% to 95%, respectively. They acquired half-body imaging from skull base to thighs 90 min post-tracer injection, but nonetheless concluded that the improvement in preoperative staging provided by ^18^F-FDG PET/CT versus CT alone was small and therefore this advantage was not significant enough to justify the additional cost. Therefore, they recommended using ^18^F-FDG PET/CT only in patients with either enlarged pelvic lymph nodes and/or extra pelvic nodal metastases or indeterminate lesions suspicious for metastasis [48]. In this sense, a retrospective study by Pichler et al. [49] assessed 70 bladder cancer patients for staging purposes with ^18^F-FDG PET/CT before RC and compared the performances in terms of sensitivity, specificity, and accuracy of FDG-PET alone, CT alone with a cut-off for suspicious pelvic lymph nodes of either 8 mm or 10 mm, and PET/CT combined. They found that combined ^18^F-FDG PET/CT using 8 mm cut-off resulted in a nonsignificant increase of diagnostic accuracy compared to CT alone (83% vs. 84% respectively), whereas when the threshold was raised to 10 mm, the increase of sensitivity became statistically significant (from 27.3% to 63.6%); therefore the authors concluded that an additional ^18^F-FDG PET/CT imaging in the preoperative setting before RC, for lymph node staging, could be recommended, only if the threshold of positive pelvic lymph nodes at CT evaluation is set at 10 mm, since this technique may not be appropriate to detect smaller (<8 mm) metastases. Crozier et al. [50] published a review and metanalysis to compare the sensitivity and specificity of different imaging modalities for staging in bladder cancer and included studies in which lymph node imaging findings were compared with final histopathology. From their results, they identified 35 articles that demonstrated a higher sensitivity for MRI and PET/CT compared to CT, whereas the specificity was similar for all modalities, hence the authors stated that in their opinion, CT will continue to be the modality of choice when staging for bladder cancer as it still has the advantages of being widely available, low cost, and with a short acquisition time. However, when considering MIBC patients or high-risk NMIBC, the recommendation may change, as demonstrated in a recent metanalysis by Soubra et al. [51]. The authors reviewed original articles, including their own study focusing on the accuracy of ^18^F-FDG PET/CT in detecting lymph node metastasis at a preoperative stage in patients with MIBC or high-risk NMIBC. The eight studies included had different patient preparation and diuresis protocols, which varied from none to saline, up to 40 mg of furosemide, although no statistically significant difference was found between studies in terms of diagnostic accuracy; they also obtained a pooled sensitivity for ^18^F-FDG PET/CT of 0.565, significantly higher than 0.35 of CT alone, and a pooled positive LR of 9.02, which could imply a change in the therapeutic approach, mainly in the case of patients with low pretest probability of positive locoregional lymph nodes and positive PET/CT scan, regardless of whether or not they have received chemotherapy. In this sense, ^18^F-FDG PET/CT demonstrated in several studies of patients with high-risk MIBC that it could provide additional staging information compared to CT alone, and that it could influence the treatment plan in approximately 20–30% of patients. Nonetheless, due to the above limitations, namely ^18^F-FDG accumulation into the bladder and excretion in the urine, which eventually would mask tracer uptake of the adjacent lymph nodes, the use of ^18^F-FDG PET/CT for staging of MIBC is not yet accepted in clinical practice and conventional imaging techniques, such as abdomen/pelvis CT or MRI, remain of choice [52] (Figure 1).

## 5. ^18^F-FDG PET/CT for Restaging and Response Evaluation of MIBC Following Neoadjuvant Chemotherapy

In contrast with the minimum improvement provided by ^18^F-FDG PET/CT in the case of staging MIBC patients, and based on literature data, this diagnostic tool can be a useful test for the detection of recurrent tumor in the pelvis and for the detection of distant metastases, thus in the case of re-evaluation after treatment, as well as to differentiate between local recurrent disease versus post-surgical or post-irradiation fibrosis or necrosis. Van de Putte et al. [53] investigated the accuracy of ^18^F-FDG-PET/CT for response evaluation following NAC, studying 37 patients that received a ^18^F-FDG PET/CT before and after NAC, followed by RC. Delayed pelvic imaging was performed according to their own standardized protocol: 90 min after FDG injection, patients received 20 mg of furosemide i.v. and an extra oral intake of 500 mL water; patients were also asked to void frequently and pelvic PET/CT imaging was then performed 3.5 h after ^18^F-FDG injection, covering a range of two bed positions centered at the location of the bladder. Their results suggest that ^18^F-FDG PET/CT is a valuable tool to accurately distinguish between primary tumor downstaging and non-response, which implies that response monitoring could be used to adjust NAC; in addition, the NPV they observed for complete response (CR) was low, especially in case of lymph nodes, which indicates that persistent disease is still presents in case of a negative nodal result from PET/CT following NAC. Therefore, they concluded that ^18^F-FDG PET/CT cannot be used to select patients for RC. Kollberg et al. [54] enrolled 50 patients with oligometastatic MIBC selected for NAC that underwent two ^18^F-FDG PET/CT examinations at baseline and after three cycles of platinum-based chemotherapy; PET findings were correlated with histological response in excised lymph nodes. They observed 43 responder patients on sequential PET images, demonstrating 86% sensitivity in nodal status prediction after treatment. More recently, a Chinese group [55] evaluated the diagnostic performance of delayed ^18^F-FDG PET/CT in the differentiation of residual tumors from postoperative inflammatory reactions; to do so, they retrospectively analyzed the data of 79 patients with BC that, after the routine whole-body ^18^F-FDG PET/CT imaging, were administrated 40 mg furosemide per os and asked to drink 1500–2000 mL of water; after about two hours only the pelvis was imaged during the second acquisition with one bed position. Their results showed that SUV mean (mean, 9.3 ± 5.4 vs. 5.8 ± 2.0), SUV max (mean, 22.2 ± 13.6 vs. 15.5 ± 9.8), and lesion thickness (mean, 17.9 mm ± 11.1 vs. 9.6 mm ± 4.1) were significantly higher in residual tumors than in inflammatory reactions, respectively. In addition, through ROC analysis, they established SUV mean greater than 8.7 and lesion thickness greater than 12.8 mm as best cut-off to differentiate residual bladder tumors from postoperative inflammatory reactions [55]. With respect to MIBC, a retrospective study of 29 patients [56], who had undergone or not cystectomy, assessed staging performance of ^18^F-FDG PET/CT with forced diuresis and delayed imaging in the setting of after treatment or follow up. In particular, additional delayed images of the pelvic region were acquired 60–90 min after IV furosemide (0.5 mg/kg body weight) and oral hydration (1000–1500 mL water) and patients were asked to evacuate the bladder frequently (at least three times). According to their results, bladder activity was reduced to background levels in 21 of 22 bladder-preserved patients, which allowed an adequate evaluation of hypermetabolic lesions in the urinary bladder as well as in the perivesical nodes. In fact, PET images allowed the identification of sixteen hypermetabolic bladder lesions, subsequently confirmed as active cancerous foci at cystoscopic biopsy, whereas CT detected wall thickening in the corresponding areas at only nine sites; thus, in seven cases, hypermetabolism was the only abnormality detected, without any wall thickening on corresponding CT images, which means that CT was false negative for early recurrence in the bladder wall in seven of 16 lesions. For this reason, the authors concluded that composite PET/CT (with post-diuretic delayed imaging) images, by providing both morphological and metabolic information, has the potential to significantly reduce the false positives of PET and CT performed separately and it should replace CT of the abdomen in the restaging protocol for recurrent invasive bladder cancers [56]. Another aspect in which ^18^F-FDG PET/CT could represent a valuable modality is in establishing patients’ prognosis. In this sense, Alongi et al. in 2016 [57] retrospectively studied 41 patients with BC that underwent ^18^F-FDG PET/CT for local recurrence or metastatic involvement by using semiquantitative PET values, such as SUV_max_, SUV_mean_, SUL, MTV, and TLG, to assess progression-free survival (PFS) and overall survival (OS) using Kaplan–Meier curves. Of the 21 patients in which PET/CT scan was considered positive, recurrent BC was confirmed in 20 (95%); their results showed an overall sensitivity, specificity, and accuracy of 87%, 94%, and 90%, respectively, with ^18^F-FDG PET/CT findings that modified the therapeutic approach in 16 patients (40%). Moreover, their data showed that PFS was significantly higher in patients with negative scans vs. those with pathological findings, and OS significantly reduced in case of positive scan, regardless of nodal or metastatic involvement, with SUV max > 6 as the most accurate threshold; thus, they concluded that ^18^F-FDG PET/CT in patients with suspected recurrent BC has a very good diagnostic performance and prognostic value [57]. These data, although quite inhomogeneous, therefore suggest the usefulness of ^18^F-FDG PET/CT for restaging and response evaluation of MIBC.

## 6. Alternative Tracers for MIBC Functional Imaging

Due to the physiological activity of ^18^F-FDG in the urinary tract, which inevitably limits the assessment of bladder region, several different tracers have been used for primary tumor detection and local staging of bladder cancer, i.e., to assess lymph node involvement, such as ^11^C-choline, ^11^C-acetate, and ^11^C-methionine. ^11^C-choline shows minimal urinary excretion with increased uptake in neoplastic lesions in the form of ^11^C-phosphorlycholine, which is trapped inside the cell [58,59,60]. ^11^C-acetate is firstly converted to acetyl-CoA, which is then converted into fatty acids and incorporated into the intracellular phosphatidylcholine membrane microdomains; ^11^C-methionine uptake instead is proportional to amino acid transport and represents an indirect estimate of protein synthesis, hence, methionine levels have been correlated with the amount of viable tumor tissue [61]. A recent literature review and meta-analysis by Kim et al. [62] assessed the diagnostic accuracy of ^11^C-choline and ^11^C-acetate PET/CT for lymph node staging in patients with BC. They included in their analysis ten studies from 2002 and 2015 for a total of 282 patients and obtained a pooled sensitivity and specificity of 0.66 and 0.89, respectively. Therefore, they concluded that both tracers show low sensitivity and moderate specificity [62]. Golan et al. [63] compared ^11^C-choline PET/CT with FDG-PET/CT in the evaluation of 51 lesions with abnormal activity in 20 patients; they found PPV for all lesions of 84% for ^11^C-cholinePET/CT and 90% for ^18^F-FDG PET/CT, whereas in the evaluation of extravesical lesions, PPV was 79% and 88%, respectively. In addition, ^18^F-FDG PET/CT could correctly identify four extravesical metastases that were missed by ^11^C-choline PET/CT, therefore they concluded that ^11^C-cholinePET/CT was not superior to ^18^F-FDG-PET/CT in detecting metastatic BC [63]. Vargas and colleagues prospectively assessed the diagnostic performance of MRI, ^11^C-acetate PET/CT, and contrast-enhanced CT (ceCT) for bladder staging in 16 patients before RC and pelvic lymph node dissection, using pelvic lymph node pathologic review as reference standard. They concluded that all techniques displayed similar levels of accuracy and that a positive history of chemotherapy reduces staging accuracy [64]. Analogously, Picchio et al. studied 27 patients with urothelial bladder cancer referred for RC and lymph node dissection with ^11^C-Choline PET and CT scan to determine the diagnostic performance in preoperative staging. While CT scan detected positive lymph nodes in 50% of patients, ^11^C-Choline PET scan identified 68% of them as positive findings, thus concluding that the latter showed significantly higher diagnostic accuracy compared to CT in detecting lymph node metastases [65]. Schöder et al. in 2012 [66] investigated the utility of ^11^C-acetate-PET/CT for the staging of MIBC and in particular they analyzed the accuracy of the technique in the assessment of response to NAC. They studied seventeen patients that underwent ^11^C-acetate-PET/CT prior to RC and pelvic lymph node dissection. It was concluded that ^11^C-acetate-PET/CT offered high sensitivity in the detection of lymph node metastases; however, inflammation and granulomatous infections and false positive results following intravesical Bacillus Calmette–Guerin therapy were reported as the limitations of this method [66]. At the moment, therefore, the data reported in the literature are too scarce to evaluate technique in these patients.

Finally, it is worth mentioning the preliminary preclinical results of ^89^Zr-DFO-HuMab-5B1, an immuno-PET tracer that selectively targets carbohydrate antigen 19.9 (CA19.9), a. useful biochemical marker of several types of cancer, including urothelial carcinoma. ^89^Zr-DFO-HuMab-5B1 is also currently in a phase I trial for pancreatic cancer expressing CA19.9, whereas preclinical results of its validity have already been published for bladder cancer by Escorcia et al. [67]. The authors demonstrated specific PET uptake in mice with subcutaneous xenografts of human bladder cancer line HT 1197 and concluded that the radioimmunoconjugate not only can detect human urothelial cancer especially in patients with elevated CA19.9 levels but it could also guide the development of targeted therapies [67].

## 7. Other Diagnostic Imaging Procedures for the Evaluation of Treatment Response

Evaluation of treatment response before surgery is crucial for the application of bladder-sparing techniques, especially in case of MIBC. Yoshida et al. [68], back in 2010, investigated the feasibility of DWI imaging and compared it to conventional MRI imaging, since conventional techniques are not capable of distinguishing between residual cancer from treatment-related changes. To do so, they evaluated 42 patients with MIBC who underwent induction low-dose chemo-radiotherapy and MRI. They reported statistically significant superiority of DWI over T2W and DCE in terms of specificity and accuracy (92% and 80%, respectively), thus concluding that DWI is useful to predict pathologic complete response, allowing optimal patient selection [68].

In 2021, Ahmed et al. [69] prospectively studied 90 patients with MIBC after NAC and set cut-off values in order to standardize the prognostic significance of the diffusional study (using the ADC map and wash-out rate). In particular, a cut-off ADC value was defined at 0.911 × 10^−3^ mm^2^/s and wash-out rate at 0.677 min^−1^ with sensitivity/specificity in predicting pathologic complete response equal to 96%/97%, respectively. Therefore, the authors concluded that DW-MRI is a potential biomarker for predicting pathologic complete response, especially in the case of the combination of wash-out parameters and ADC [69].

MR lymphography (MRL) represents another possible approach in this specific clinical setting since the utility of MRL for bladder cancer lymph node staging has been investigated by several study groups, in particular using ultra-small superparamagnetic iron oxide (USPIO) nanoparticles as a contrast medium. In 2004, Deserno et al. [70] showed the significant improvement in terms of sensitivity, specificity, and NPV for lymph node involvement provided by MRL compared to MRI alone. They prospectively enrolled 58 patients scheduled for RC that underwent MR imaging before and after USPIO injection and compared the results with histopathologic analysis of surgically removed lymph nodes. They reported an increase in sensitivity from 76% to 96% and negative predictive value from 91% to 98% (*p* < 0.01) [70]. However, the main downsides of MRL are the amount of time needed for study completion (up to 36 h) and the difficulty of images interpretation [71]. More recently, Birkhäuser et al. prospectively evaluated the diagnostic accuracy of the combination of USPIO enhancement and DW-MRI for lymph node staging in bladder and/or prostate cancer, for a total of 75 patients defined as N0 by conventional cross-sectional imaging [72]. They used pelvic lymph node dissection as reference standard and reported 65–75% sensitivity and 93–96% specificity with a median reading time for the combined USPIO-DW-MRI images of nine minutes. Therefore, they concluded that this technique improves detection of metastases in normal-sized pelvic lymph nodes, in at least two-thirds of their patients [72].

## 8. Hybrid Imaging with PET-MRI

Hybrid PET-MRI imaging combines functional data of PET with the anatomical high image quality of MRI, thus providing great contrast of soft tissue [73]. Catalano et al. compared the diagnostic performance of PET/CT and PET/MRI in an unselected heterogeneous population of 134 patients with various types of cancer and reported that findings were fully concordant in 73 patients (54.5%), however PET/MRI revealed additional findings in 55 patients (41%), which were not seen in the PET/CT [74]. As concerning pelvic malignancies, they therefore concluded that PET/MRI could overcome intrinsic limitation of PET/CT in the assessment of local extent of the disease [74].

Analogously, a recent paper analyzed 22 patients with bladder cancer in a prospective pilot study in order to compare the diagnostic performance of MRI alone versus FDG PET/MRI using a diuresis protocol based on the administration of intravenous furosemide and oral hydration. PET/MRI showed higher accuracy (95% vs. 76%) in the detection of metastatic pelvic lymph nodes, providing more accurate staging mainly in case of equivocal findings of MRI alone [75].

Eulitt et al. From the University of North Carolina, in a recent pilot study on twenty-one patients with planned radical cystectomy, concluded that novel imaging modalities, such as FDG-PET/MRI, may improve the diagnostic accuracy for the staging of bladder cancer [76].

Salminen et al. evaluated the accuracy of ^11^C-acetate PET/MRI in 15 patients with BC for staging purposes and to monitor responses to NAC. In the case of MIBC ^11^C-acetate PET/MRI showed 100% sensitivity, 69% specificity, and 73% accuracy, although they reported an overall low sensitivity (approximately 20%) for the detection of nodal metastases [77].

## 9. Conclusions and Future Perspectives

Bladder cancer has been considered for a long time a solid tumor in which ^18^F-FDG and PET could not give a significant contribution. This assumption was supported by technical and biological drawbacks due to FDG bio-distribution; ^18^F-FDG renal excretion in the past therefore suggested it a suboptimal tracer for detecting a tumor in the bladder wall, even in the detection of lymph node metastases. The clinical scenario was mainly surgical oriented and did not require further imaging other than cystoscopy, CT, and MRI.

The improvements of PET-CT performances and of patient preparation, together with a wide use of NAC or biological treatments, are now changing the perceptions of urologists and oncologists regarding molecular imaging. We are fascinated by the chance to tailor a treatment on the basis of images that expresses tumor biology in a whole body scan in a reliable manner. All these aspects have changed FDG from a suboptimal tracer to a promising tool in MIBC patients.

Hybrid PET/MR imaging has demonstrated encouraging results but limited availability limits to date its use in daily management of MIBC patients.

Future directions include the opportunity to connect biological data and imaging to predict treatment sensitivity by using artificial intelligence (AI) techniques. AI, radiomics, and routine PET images can be transformed into useful data analyzed by a “trained” machine, hence with high reproducibility, together with clinical, genetic, and molecular information, to provide tailored diagnosis and treatment. These innovative approaches seem to be affordable in clinical practice more than innovative tracers.
